# Editorial: Aging: challenges and opportunities for inclusion and active participation

**DOI:** 10.3389/fpsyg.2025.1725888

**Published:** 2025-12-01

**Authors:** Ramona Bongelli, Chiara Aleffi, Alessandra Fermani, Veronica Guardabassi, Gonzalo del Moral Arroyo, Paola Nicolini

**Affiliations:** 1 Department of Political Science, Communication and International Relations, University of Macerata, Macerata, Italy; 2 Department of Education, Cultural Heritage and Tourism, University of Macerata, Macerata, Italy; 3 Department of Humanities, University of Macerata, Macerata, Italy; 4 Department of Education and Social Psychology, University of Pablo de Olavide, Seville, Spain

**Keywords:** active aging, inclusion, social participation, digital technologies, wellbeing

## 1 Introduction

In recent decades, the proportion of the population over the age of 65 has increased and, according to United Nations projections, it will reach 16% of the global population by 2050. While this trend reflects improved living conditions in many parts of the world, it also entails a growing prevalence of frailty and, consequently, a greater demand for care, thereby placing significant pressure on social and healthcare systems. Though aging is a heterogeneous process with significant variations between individuals, it is often associated with an increased risk of developing chronic conditions such as diabetes, respiratory diseases, neurological disorders, cardiovascular diseases, as well as cognitive impairments and decline. This frequently results in a growing need for long-term care, often provided by family members and professional caregivers. In other words, population aging increases the demand for, and the cost of, social and healthcare services, making it even more urgent to ensure that older adults receive timely, targeted care that preserves—as long as possible—their autonomy and wellbeing, while fostering genuine social inclusion and enabling full participation in society. Defining *social inclusion* is challenging, because it is a complex concept, that includes numerous components. Some definitions focus on the sense of belonging and recognition within a group, while others place greater emphasis on the procedural, relational, and action-related dimensions. Thus, *social inclusion* has been defined as “belonging to, identifying with, and feeling included in important and valued social groups (e.g., friendship groups, support groups, work groups, recreation groups)” (Hutchison and Ewens, 2022, p. 2162), as well as “a process that enhances opportunities for social participation, strengthens social bonds, and ensures equitable access to opportunities and decision-making” (Tan et al., 2025, p. 1). *Social participation* can, instead, be conceived as the concrete opportunity to act, to take part in a social and relational context. It indeed refers to individuals' engagement in activities that foster interaction with other people, taking place within community settings and shared social spaces. Obviously, this involvement evolves over time depending on available personal resources and individuals' perceptions of what is valuable and meaningful. It is also shaped by the opportunities offered by the broader social context (Levasseur et al., 2022).

## 2 The Research Topic

In December 2024, the Research Topic entitled “*Aging: challenges and opportunities for inclusion and active participation*” was launched, inspired by the VITALITY project (Innovation, Digitalisation and Sustainability Ecosystem for the Widespread Economy in Central Italy), funded by the PNRR (National Recovery and Resilience Plan). One of the overarching aims of VITALITY was to improve sustainability and quality of life in both urban and rural areas. Within this framework, the University of Macerata focused specifically on Smart solutions and educational programmes for antifragility and inclusivity (SAFINA). “Antifragility is beyond resilience or robustness. The resilient resists shocks and stays the same; the antifragile gets better” (Taleb, 2014, p. 3). It enables individuals to benefit and develop not despite, but rather, because of radical uncertainty and adversity (Klisanin, 2022). This capacity is based on interdependence, since “our psyche can withstand shocks and get stronger because we are not isolated” (Klisanin, 2022, p. 338). For older adults, this means overcoming uncertainty, limitations and challenging (e.g., psychical, psychological) that often characterize later life, through active engagement with their social environment. Crucially, antifragility in later life can only be fully developed within inclusive contexts that provide opportunities for participation, recognition, and meaningful social interactions, enabling older individuals to strengthen their adaptive and transformative capacities.

Thus, the main aim of this Research Topic was to identify, in different contexts, challenges and opportunities to address the needs of this population, as well as that of those who care for them in situations of frailty. More specifically, the goal was to gather contributions that would pay attention to the impact of aging (primarily in psychological, social, relational, and communicative terms, but not only) on older adults, caregivers and the wider community (e.g., Bongelli et al., 2024; Guardabassi, 2025; Santini et al., 2025).

For this Research Topic—the first volume of a two-volume collection—we received many highly interesting papers. Ten of them (nine original papers and one study protocol), authored by 42 researchers from different countries, with a particularly strong representation from China, were successfully published. Specifically, six papers were published in Frontiers in Public Health (sections Aging and Public Health, Life-Course Epidemiology and Social Inequalities Health, Digital and Public Health) and four in Frontiers in Psychology (sections Health Psychology and Psychology and Aging).

Although the articles address diverse topics, they can be grouped into three main thematic areas:

1. *Digital technologies and active aging;*
2. *Social participation, wellbeing, and mental health;*
3. *Cognitive stimulation and leisure activities for healthy aging*.

### 2.1 Digital technologies and active aging

The first thematic area includes papers exploring how internet use, social media engagement, smart products, and digital cultural adaptation can promote social participation, psychological wellbeing, and health among older adults. While some of them demonstrate physical and psychological benefits of internet, digital, smart, and social media use among older adults, others reveal persistent barriers, highlighting the need for targeted strategies in support, design, and services to maximize their participation and digital inclusion.

Kong and Zhu, analyzing data from the Chinese Longitudinal Aging Social Survey (CLASS) 2020, found that comprehensive use of internet (i.e., extending beyond simply entertainment) significantly reduced older adults' subjective age, which, in turn, positively affected perceived health, self-efficacy, and social capital.

Similarly, Liu et al. showed that active social media use foster physical activity among older adults in Shandon province (China), despite age-related physical decline. Taking together, these findings suggest that *active* engagement with internet and social media can help mitigate age-related decline, supporting healthier and more active aging.

Yun et al. focused instead on the emotional needs of older people living alone in urban China, and on their complex relations with smart technology, conducting, and analyzing 20 interviews. Their thematic analysis highlighted that many older persons resorted to physical, social, and spiritual activities to cope with anxiety, loneliness, and diminished autonomy, but they reported a limited use of smart products due to low motivation, usability barriers, and a lack of age-sensitive design.

In line with the previous study, also Li et al. examines the obstacles faced by people over 60 in fully participating in the digital world. Although participants reported satisfaction with digital life, personal adaptation, and content diversity, they also expressed dissatisfaction with family guidance, age-friendly digital services, and the role of digital content in improving quality of life.

### 2.2 Social participation, wellbeing, and mental health

The second thematic area includes papers on how social capital, recreational activities (including the use of the internet and social media), and social relationships influence depression, happiness, and life satisfaction among older adults, with particular attention to mediating mechanisms such as self-perceived aging and life-course experiences.

Cheng et al., through the lens of life course theory, conducted a qualitative study with 16 older adults in China, showing how past experiences of war, famine and political reform have shaped the participants' current wellbeing and life perspectives. The findings highlight that the life wisdom and happiness of Chinese seniors unfold across three main dimensions: *personal* (rooted in education, family bonds and active social participation); *social* (characterized by an ambivalent perception, combing gratitude for social welfare with concerns about contemporary issues, especially corruption); *temporal* (linked to expectations for younger generations, with a strong sense of responsibility for guiding them, and an emphasis on resilience and the transmission of accumulated wisdom). Overall, the study reveals that older adults transform early-life experiences of suffering into a form of life wisdom, which not only contributes to their own wellbeing, but is also transmitted to younger generations, thereby reinforcing cultural continuity.

Ye et al. focused instead on the role of social capital in psychological wellbeing. They conducted a cross-sectional study of 1,809 community-dwelling older adults in Chengdu and found that levels of depression not only varied according to certain socio-demographic variables (age, marital status, chronic illness, insurance coverage, and income), but that social capital was negatively associated with both self-perceived aging and depression. These results suggest that strengthening social capital—through initiatives such as senior universities, dance and chorus groups, or community sports—and fostering positive perceptions of aging may help to prevent depression.

Also Tao et al.—analyzing data from three periods of the China Health and Aged Care Tracking Survey (CHRLS), collected between 2015 and 2020 on 3,762 adults aged 60 and above—found that both leisure-oriented social activities, as well as internet use have significant protective effect against depression in older adults.

Similarly, Xia et al., analyzing data from the China Health and Retirement Longitudinal Study (CHARLS), which covered 36,934 adults aged 45 and over, found that playing Mahjong or Bridge was linked to higher subjective levels of wellbeing, specifically among those subjects (retired individuals, women, and participants from rural areas and eastern regions of the country) who engaged in these activities more frequently. Promoting diverse cultural and sporting activities within older adult communities could foster socialization, prevent cognitive decline, and ultimately enhance wellbeing.

These studies all emphasize the importance of fostering social relationships and community involvement among older adults. Participating in cultural, recreational, and digital activities, as well as feeling like an active part of a community, is essential for reducing the risk of depression and promoting an active and fulfilling life, even in later years.

### 2.3 Cognitive stimulation and leisure activities for healthy aging

The third thematic area encompasses studies on training programme and leisure activities designed to enhance cognitive and emotional wellbeing in older populations, including those with intellectual disabilities.

Cavaggioni et al. present a detailed protocol for studying the effects of a visual training programme on cognitive performance and physical fitness in individuals with intellectual disabilities (ID), hypothesizing greater improvements in cognitive and motor performance compared to usual care. They also expect the protocol to serve as a basis for developing exercise prescription guidelines for individuals with ID, offering therapists a practical and innovative approach, with the main aim of counteracting age-related physical and cognitive decline while promoting healthy aging and social participation.

Guardabassi et al. examined the role of board games in facilitating successful aging. They tested the hypothesis that wellbeing experienced during gameplay is higher than overall daily-life wellbeing, particularly when the games are of low difficulty. To this end, they conducted gaming sessions of varying difficulty with a sample of 132 older adults, divided into groups of four or five, and administered two questionnaires: the first, before each session, assessed general wellbeing; the second, after the session, assessed wellbeing during gameplay. The findings, which confirmed their hypothesis, showed that board games can be considered as a low-cost and easily adoptable intervention to enhance the wellbeing of older adults and to promote intergenerational activities in diverse settings (e.g., families, community centers).

Both studies emphasize the importance of structured, accessible activities, such as visual training or recreational gaming, as a way of sustaining cognitive, physical and emotional wellbeing in aging populations. They demonstrate that tailored, low-cost interventions can mitigate age-related decline and foster an active, meaningful aging process.

## 3 Conclusion

Although the articles in Volume I of this Research Topic cover diverse topics, a common theme emerges: active aging is closely linked to a sense of belonging to a community (social inclusion) and to social participation, through involvement in recreational, educational, sporting and cultural activities, both in person and online via the internet and digital technologies. Perceiving oneself as an integral part of and taking an active role in a community, both offline and online, appears to be a key factor in enhancing psychological wellbeing, reducing depression and counteracting negative perceptions of the aging process, promoting simultaneously antifragility.

While the contributions collected in this Research Topic highlight a series of conditions that can promote active aging, they also emphasize the persistence of significant obstacles to its achievement, including *ageism* (i.e., “the tendency to be prejudiced against older adults, to negatively stereotype them—e.g., as unhealthy, helpless, or incompetent-, and to discriminate against them, especially in employment and health care,” APA Dictionary), *socio-economic inequalities*, the *intergenerational gap* and the *digital divide*. Addressing these challenges requires integrated strategies, that combine educational, social and cultural interventions.
